# The Psychological Impact of the COVID-19 Outbreak on Health Professionals: A Cross-Sectional Study

**DOI:** 10.3389/fpsyg.2020.01684

**Published:** 2020-07-10

**Authors:** Emanuele Maria Giusti, Elisa Pedroli, Guido E. D'Aniello, Chiara Stramba Badiale, Giada Pietrabissa, Chiara Manna, Marco Stramba Badiale, Giuseppe Riva, Gianluca Castelnuovo, Enrico Molinari

**Affiliations:** ^1^Department of Psychology, Catholic University of Milan, Milan, Italy; ^2^Psychology Research Laboratory, Istituto Auxologico Italiano IRCCS, Milan, Italy; ^3^Applied Technology for Neuro-Psychology Lab, IRCCS Istituto Auxologico Italiano, Milan, Italy; ^4^Department of Psychology, eCampus University, Novedrate, Italy; ^5^Department of Geriatrics and Cardiovascular Medicine, IRCCS Istituto Auxologico Italiano, Milan, Italy

**Keywords:** COVID-19, burnout, depression, anxiety, post-traumatic stress, predictors, clinical psychology, cross-sectional study

## Abstract

**Background:** The COVID-19 pandemic had a massive impact on health care systems, increasing the risks of psychological distress in health professionals. This study aims at assessing the prevalence of burnout and psychopathological conditions in health professionals working in a health institution in the Northern Italy, and to identify socio-demographic, work-related and psychological predictors of burnout.

**Methods:** Health professionals working in the hospitals of the Istituto Auxologico Italiano were asked to participate to an online anonymous survey investigating socio-demographic data, COVID-19 emergency-related work and psychological factors, state anxiety, psychological distress, post-traumatic symptoms and burnout. Predictors of the three components of burnout were assessed using elastic net regression models.

**Results:** Three hundred and thirty health professionals participated to the online survey. Two hundred and thirty-five health professionals (71.2%) had scores of state anxiety above the clinical cutoff, 88 (26.8%) had clinical levels of depression, 103 (31.3%) of anxiety, 113 (34.3%) of stress, 121 (36.7%) of post-traumatic stress. Regarding burnout, 107 (35.7%) had moderate and 105 (31.9%) severe levels of emotional exhaustion; 46 (14.0%) had moderate and 40 (12.1%) severe levels of depersonalization; 132 (40.1%) had moderate and 113 (34.3%) severe levels of reduced personal accomplishment. Predictors of all the three components of burnout were work hours, psychological comorbidities, fear of infection and perceived support by friends. Predictors of both emotional exhaustion and depersonalization were female gender, being a nurse, working in the hospital, being in contact with COVID-19 patients. Reduced personal accomplishment was also predicted by age.

**Conclusions:** Health professionals had high levels of burnout and psychological symptoms during the COVID-19 emergency. Monitoring and timely treatment of these conditions is needed.

## Introduction

At the end of 2019, the coronavirus disease (COVID-19) first appeared in China, in particular in Whang City, in Hubei province (Wang et al., 2020a). In March 2020, due to the global spread of the disease, COVID-19 was declared as a pandemic, causing widespread concern (World Health Organization, 2020b). In fact, COVID-19 is an international public health emergency unprecedented in modern history and it causes several health and psychological problems among general population including high level of anxiety, depression and stress (Ornell et al., 2020).

As of the first half of May, 2020, Italy was one of the most affected countries during this outbreak, counting over 223,000 individuals infected by COVID-19 and more than 31,000 casualties (World Health Organization, 2020a). The high prevalence of the disease in the Northern regions of the country led to a national reorganization of the hospital network and caused sudden changes in the personal and professional lives of healthcare professionals.

Epidemic studies proved that previous infectious diseases caused long-term and persistent psychopathological consequences among this category (Tam et al., 2004; Lee et al., 2007). For example, during and after the Severe Acute Respiratory Syndrome (SARS) outbreak in 2003, frontline healthcare professionals self-reported lack of support in the workplace and consequently severe psychological symptoms as acute distress (Tam et al., 2004). Also, in 2015, during the Middle East respiratory syndrome (MERS) outbreak, the medical staff showed an increased long-term risk of developing post-traumatic stress disorder (PTSD), leading to a boost in absenteeism from work (Lee et al., 2018). SARS and MERS experiences crucially compromised healthcare professionals' well-being. In fact, during epidemic emergencies, as it is happening during COVID-19, frontline care workers experience an unexpected increase in workload in a context of uncertainty and powerlessness, and are more vulnerable to the infection due to their direct contact with patients, which also increases in turn their concerning about infecting their families and colleagues (Liu et al., 2020; Ran et al., 2020). As of the end of April 2020, about 12,000 between doctors and nurses were infected by COVID-19, and 228 doctors and 26 nurses had died (Fusaroli et al., 2020; Manzoni and Milillo, 2020). Non-frontline healthcare workers are also at risk for increased stress due to reduced accessibility to formal psychological support, less first-hand medical information on the outbreak, less intensive training on personal protective equipment and infection control measures (Tan et al., 2020a).

During pandemics, general population have been safeguarded with several precautionary measures including shutdown or slowdown in daily activities, social distancing, reductions in interactions between people, wearing face masks and have good ventilation to reduce the possibility of new infections (Tan et al., 2020b; Wang et al., 2020b; Wilder-Smith and Freedman, 2020). On the contrary, healthcare professionals were exposed to longer work shifts, in order to manage the growth of health care demand (Huang et al., 2020; Ornell et al., 2020). These critical conditions are exacerbated by the need of wearing personal protective equipment which cause discomfort and difficulties in breathing.

At the beginning of the spreading of the virus, hospitals had limited availability of personal protective equipment and guidelines or treatment were not well-established (Xiang et al., 2020). Therefore, many professionals felt confused and unprepared to treat adequately patients infected by the new virus (Huang et al., 2020). As a consequence, they perceived feelings of uncertainty, helplessness, alienation, isolation and difficulties in managing the workload. Furthermore, operators had to face loneliness, perception of stigma and rigid expectations, which can lead to several emotional and psychological outcomes as anger, anxiety, insomnia, and stress related to the uncertainty of the outbreak (Ran et al., 2020; Zhang et al., 2020). All the above-mentioned risk factors can induce more likely the onset of burnout (Ornell et al., 2020).

Burnout can be defined as a psychological syndrome characterized by chronic exhaustion, cynicism and ineffectiveness and it emerges as a response to the presence of highly stressful conditions in the workplace (Maslach and Goldberg, 1998). The presence of burnout among health operators, in particular but not limited to doctors and nurses, has a very strong impact both on their physical and psychological health and on the efficiency of their organization and work (Portoghese et al., 2014; Low et al., 2019; Woo et al., 2020). Usually, burnout occurs following long-term exposition to organizational risk factors but critical emergences, like pandemics, can easily trigger emotional exhaustion (Kim and Choi, 2016).

During pandemics or other critical situations, protective factors can help healthcare professionals to cope with the emergency. For instance, after SARS, health professionals reported that clear directiveness and support from the supervisors, adequate training, precautionary measures, social, religious, and familiar support were the most effective coping strategies (Chan and Huak, 2004; Cheng and Wong, 2005; Maunder et al., 2006). Personality traits also proved to influence health professionals' responses to the pandemics. In fact, during SARS emergence, optimism, resilience and altruism reduced psychological distress among healthcare workers (Bai et al., 2004; Lee et al., 2007).

Furthermore, after MERS, medical staff sustained that several factors including strict protective measures and guidance, the presence of a cohesive team, positive attitudes in the workplace and the recognition of their efforts by the hospital helped them to face the situation (Khalid et al., 2016).

Recent scientometric analysis found that the most common research topics include emergency care and surgical, viral pathogenesis, and global responses in the COVID-19 pandemic but there is a lack of mental health research and only few studies addressed the impact of the COVID-19 pandemic on healthcare professionals' well-being (Tran et al., 2020). Therefore, the main objective of this study is to identify the prevalence of burnout and psychological distress in health professionals during the early phases of the pandemic. The secondary objective of this study is to assess the demographic, psychological, and work-related predictors of burnout.

## Materials and Methods

This study is part of a broader project, the COV-BHP study, which is a prospective cohort study aimed at identifying the prevalence and predictors of burnout and psychological distress in health professionals working in the Hospitals of the Istituto Auxologico Italiano. The Istituto Auxologico Italiano is a scientific and clinical institution operating with three main hospitals in Lombardy and Piedmont (Northern Italy), the regions with the higher transmission rates and mortality in Italy (Dipartimento della Protezione Civile, 2020). About 800 health professionals work in this Institution. All of them were informed about the study through an institutional e-mail message, which also reported an anonymous link that enabled to be enrolled in the study after giving an informed consent. The e-mail was sent on April 16, 2020 and data collection was discontinued on May 11, 2020. The whole study was performed using online questionnaires implemented using the Qualtrics software, version 03/2020 (Qualtrics, Provo, UT). The study was approved by the Institutional Ethical Committee.

### Measurement Instruments

Data collected in the survey included:

- Socio-demographic and clinical factors: gender, age, occupation, current working situation (full-time working in the Hospital, part-time working in the Hospital, working from home, being quarantined), medical or psychopathological comorbidities;
- COVID-19 emergency-related work factors: number of hours per week spent working, exposure to COVID-19 cases (no exposure, exposure to suspect COVID-19 cases, exposure to confirmed COVID-19 cases), working in wards dedicated exclusively to the care of patients with COVID-19 patients, number of days since the professional's working situation changed because of COVID-19;
- COVID-19 emergency-related psychological factors: single items measured through a cursor on a pointed scale from 0 to 100, adapted from a previous study on MERS epidemic (Kim and Choi, 2016). COVID-19 emergency-related psychological factors included fear of COVID-19 infection due to work-related exposure (“I am afraid of being infected with COVID-19 since I deal with COVID-19 patients”), perceived support from family and friends (“My family supports me even if my work carries risks of infection,” “My friends supports me even if my work carries risks of infection”)of for caring for COVID-19 patients”); chances to find spiritual comfort (“In facing the COVID-19 crisis, I find comfort in spirituality”);
- State Anxiety: State-Trait Anxiety Inventory—State form (STAI-S) (Spielberger et al., 1983). The STAI-S measures participant's state anxiety, i.e., the transitory state of fear and emotional tension as a response to a perceived threatening situation. The STAI-S includes 20 items on a 4-points Likert scale (not at all, somewhat, moderately so, very much so), with higher values indicating higher state anxiety. Examples of its items are “I am tense” and “I am worried.” The cut-off value of 40 was employed to identify participants with clinical levels of anxiety (Spielberger et al., 1983). This scale has been widely validated and its Italian translation has shown good psychometric properties (Pedrabissi and Santinello, 1989);
- Psychological distress: Depression, Anxiety and Stress Scale-21 (DASS) (Lovibond and Lovibond, 1995). This scale includes 21 items measured on a 4- points Likert scale (never, sometimes, often, almost always) which measure the three psychological subdimensions of psychological distress, namely anxiety (e.g., “I felt I was close to panic”), depression (e.g., “I felt that I had nothing to look forward to”) and stress (e.g., “I found it difficult to relax”). Higher values indicate higher psychological distress. Clinical levels of depression, anxiety and stress were detected identifying values above the 75° percentile based on normative data (Henry and Crawford, 2005). This scale has been validated in Italian and provides reliable and valid measurements of psychological distress in health workers (Bottesi et al., 2015);
- Post-traumatic symptoms: Impact of Event Scale—Revised-−6 items version (IES-6) (Weiss, 2007). The IES-6 is a reduced version of the 22-items IES-R instrument. It is a self-report questionnaire assessing psychological distress in response to a traumatic event. It includes 3 subscales, representing symptoms clusters of post-traumatic stress: intrusion (e.g., “I thought about it when I didn't mean to”), avoidance (e.g., “I tried not to think about it”) and hyperarousal (e.g., “I felt watchful or on guard”). Respondents are asked to indicate on a 5-point Likert scale ranging from never (score 0) to often (score 4) how frequently each symptom was experienced during the past week. The cut-off of 9 was used to dichotomize the total score (Thoresen et al., 2009). The Italian translation showed psychometric features similar to the original version (Giorgi et al., 2015);
- Burnout: Maslach Burnout Inventory (MBI) (Maslach et al., 1997). The MBI is a 22-items questionnaire on a 5-points Likert scale which assesses the three theoretical components of burnout syndrome, namely emotional exhaustion (“I feel emotionally drained from my work,” depersonalization (“I feel I treat some patients as if they were impersonal objects”) and personal accomplishment (“I deal very effectively with the problems of my patients). Higher scores in the emotional exhaustion and depersonalization scales indicate greater burnout, whereas higher scores in the personal accomplishment subscale indicate less burnout. Cutoffs for moderate and severe emotional exhaustion were ≥17 and ≥27, for moderate and severe depersonalization ≥7 and ≥13, and for moderate and severe reduced personal accomplishment ≤38 and ≤21 (Maslach et al., 1997). The MBI Italian translation has been validated for its use on health workers (Sirigatti et al., 1988).

### Statistical Analysis

Descriptive statistics are presented as counts and proportions for categorical variables and means and standard deviations for continuous variables. For descriptive purposes, scores of the burnout and psychological distress questionnaires were categorized using the appropriate cut-offs. Associations between categorical variables and burnout components were assessed using point-biserial correlations, whereas associations between continuous variables and burnout components calculating Person's r coefficient. Predictors of the burnout components, analyzed as continuous variables, were then assessed using elastic net linear regressions. Briefly, elastic net regression is a penalized linear regression analysis technique which enables to address multicollinearity between the predictors and to select the most important ones. This is done by regularizing (shrinking) their estimated β coefficients applying a penalization based on two hyperparameters. The first hyperparameter is α, which identifies the type of penalty, which ranges from a ridge penalty (based on the squared magnitude of the coefficients) when α approaches 0 to a lasso penalty (based on the absolute magnitude of the coefficients) when α approaches 1. The second hyperparameter is λ, which identifies the amount of penalization (Zou and Hastie, 2005).

We used 10-fold repeated (10 times) cross-validation to train and tune our model over a grid of α and λ hyperparameters on half of the sample, which constituted the training dataset. The model was refit on the training dataset with the best performing hyperparameters to calculate the final penalized β coefficients. The model was then applied to the other half of the sample, which constituted the testing dataset, to calculate model performance. The above procedure was repeated for each of the three dependent outcome variables. Elastic net regression was performed using the R (version 3.5.1) packages *caret* (Kuhn, 2015) and *glmnet* (Friedman et al., 2010).

## Results

### Description of the Sample and Prevalence of Psychological Symptoms

Three hundred and thirty out of the about 800 health professionals working in the Institution participated to the online survey. Table 1 reports the demographic, work-related and psychological characteristics of the participants of this research.

**Table 1:** Demographic, work-related, and psychological characteristics of the study participants.

**Variable**	***N***	**%**	**Mean**	**SD**
**Gender**				
Male	124	37.4		
Female	206	62.6		
Age			44.6	13.5
**Occupation**				
Doctor	140	42.2		
Nurse	86	26.0		
Nurse assistant	38	11.5		
Physiotherapist	35	10.6		
Other	32	9.7		
**Work status**				
Working in the hospital	232	70.3		
Working from home or being quarantined	98	29.7		
**Working in contact with COVID-19 patients**				
Yes	238	72.2		
No	92	27.8		
**Working in a COVID-19 ward**				
Yes	188	56.8		
No	142	43.2		
Work hours during the last week			25.8	16.8
**Having been infected by COVID-19**				
Yes	88	26.5		
No	242	73.5		
**Medical comorbidities**				
Yes	80	24.2		
No	250	75.8		
**Psychological comorbidities**				
Yes	12	3.6		
No	318	96.4		
Fear of infection (range 0–100)			53.3	33.9
Support from family (range 0–100)			81.1	31.1
Support from friends (range 0–100)			74.2	32.9
Support from spirituality (range 0–100)			38.2	36.4
MBI—Emotional exhaustion (range 0–54)			22.3	11.4
MBI—Depersonalization (range 0–30)			4.7	5.4
MBI—Personal accomplishment (range 0–48)			33.7	6.8
STAI—State anxiety (range 20–80)			47.3	11.9
DASS-21—Anxiety (range 0–21)			3.3	3.6
DASS-21—Depression (range 0–21)			4.0	4.2
DASS-21—Stress (range 0–21)			6.8	4.8
IES-6—Intrusion (range 0–8)			4.0	2.3
IES-6—Avoidance (range 0–8)			2.8	1.9
IES-6—Hyperarousal (range 0–8)			3.2	2.1

*MBI, Maslach Burnout Inventory; STAI, State-Trait Anxiety Inventory; DASS-21, Depression Anxiety Stress Scales; IES-6, Impact of Event Scale-6*.

**Figure 1:**
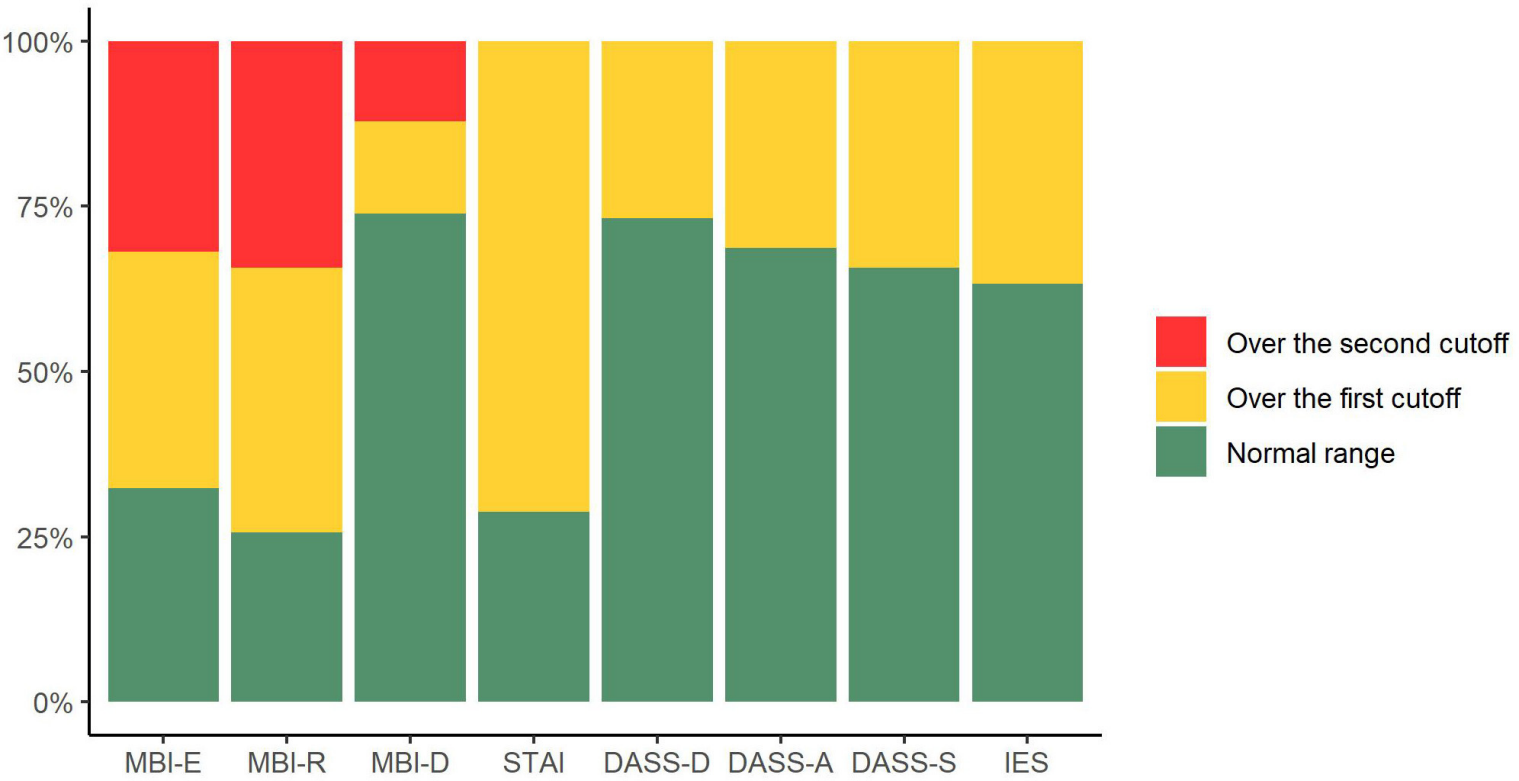
Prevalence of burnout, state anxiety, trait anxiety, depression, stress, and post-traumatic symptoms in health professionals participating to the study. MBI-E, Maslach Burnout Inventory-Emotional Exhaustion; MBI-R, Maslach Burnout Inventory-Reduced personal accomplishment; MBI-D, Maslach Burnout Inventory-Depersonalization; STAI, State-Trait Anxiety Inventory; DASS-D, Depression Anxiety Stress Scales 21-Depression; DASS-A, Depression Anxiety Stress Scales 21-Anxiety; DASS-D, Depression Anxiety Stress Scales 21-Stress; IES, Impact of Event Scale-6.

Figure 1 represents the prevalence of burnout and psychological distress in the participants. Two hundred and thirty-five health professionals (71.2%) had state anxiety scores above the cutoff. Regarding burnout, 107 (35.7%) had moderate and 105 (31.9%) severe levels of emotional exhaustion; 46 (14.0%) had moderate and 40 (12.1%) severe levels of depersonalization; 132 (40.1%) had moderate and 113 (34.3%) severe levels of reduced personal accomplishment. Clinical levels of depression were identified in 88 participants (26.8%), clinical levels of anxiety in 103 (31.3%) and clinical levels of stress in 113 participants (34.3%). Finally, 121 (36.7%) reported symptoms of post-traumatic stress.

### Predictors of Burnout

**Figure 2:**
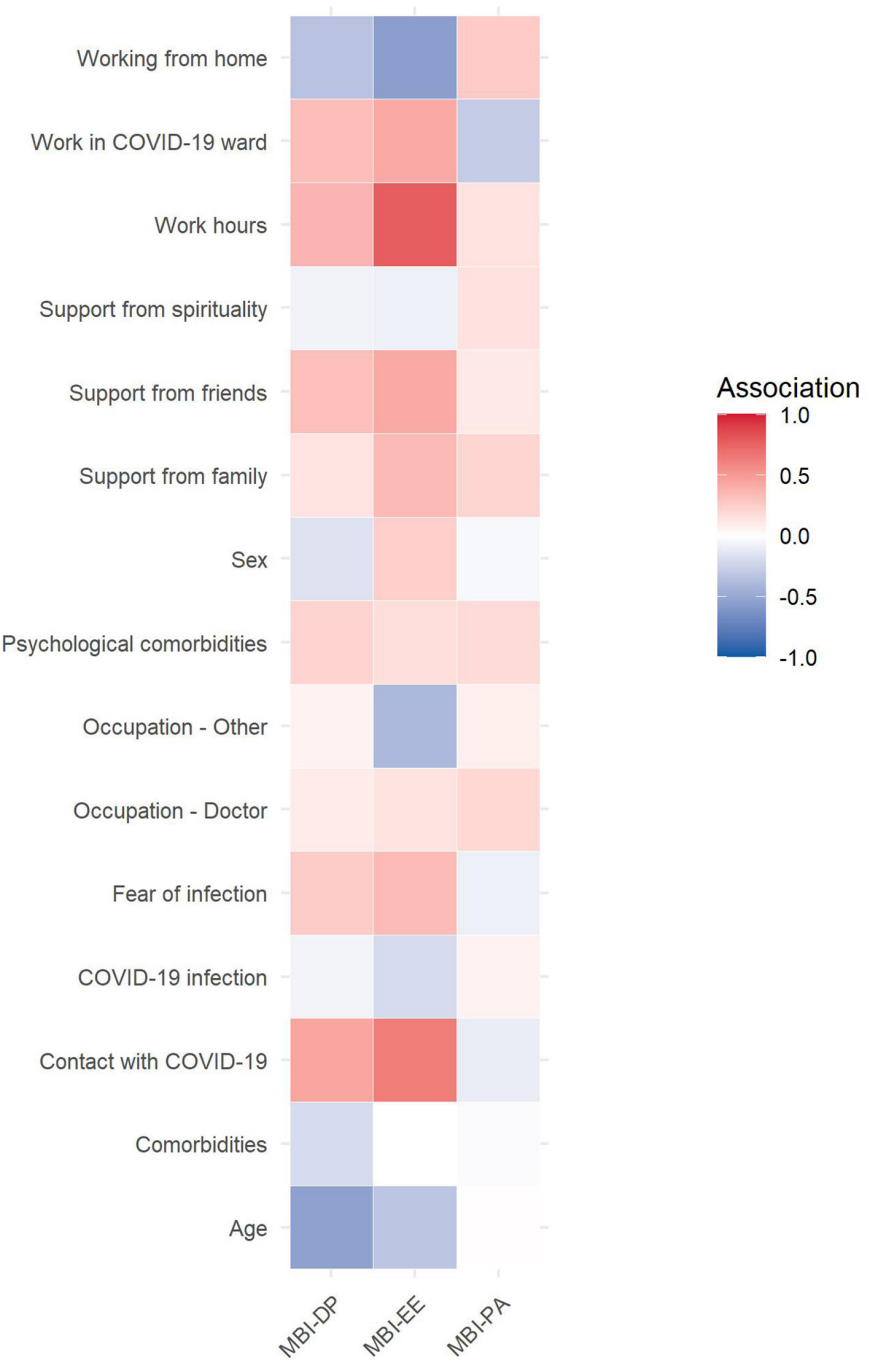
Heatmap of the association between socio-demographic, COVID-19-related, and psychological factors and burnout components. Note. Association between categorical variables and burnout were assessed using point-biserial correlation, association between continuous variables and burnout calculating Pearson's r coefficients.

The associations between categorical and continuous predictors and burnout components are plotted in Figure 2. Variables with association coefficients > 0.3, corresponding to a weak or moderate effect, were age, occupation, being home, work hours, psychological comorbidities, contact with COVID-19 patients, fear of infection, support from family and support from friends.

Finally, elastic net regressions were performed. After model tuning, the best hyperparameter for the Emotional Exhaustion subscale were α = 0.21 and β = 0.17; for the Depersonalization subscale were α = 0.28 and β = 0.17; for the Personal Accomplishment subscale were α = 0.16 and β = 0.60. Selected predictors and their coefficients are reported in Table 2.

**Table 2:** Results of the elastic net regression models investigating the predictors of the components of burnout.

	**Emotional exhaustion**	**Depersonalization**	**Reduced personal accomplishment**
	**β^a^**	**β^a^**	**β^a^**
Female gender	0.04	0.05	
Age			0.10
Occupation—Doctor (vs. nurse)			
Occupation—Other (vs. nurse)	−0.05	−0.07	
Work status—working from home or being quarantined (vs. working in the hospital)	−0.06	−0.07	
Work hours	0.20	0.18	−0.04
**Presence of medical comorbidities**			
Presence of psychological comorbidities	0.11	0.03	−0.01
Having been infected by COVID-19			
Being in contact with COVID-19 patients	0.08	0.09	
**Working in a COVID-19 ward**			
Fear of infection	0.14	0.14	−0.04
Support from family			
Support from friends	−0.05	−0.06	0.05
Support from spirituality			
Prediction *R*^2^	0.13	0.14	0.11

*Predictors were standardized before the analyses. All coefficients are penalized by the elastic net. Coefficients were computed using half of the sample and prediction R^2^ using the other half of the sample. Coefficients shrunk to 0 are not displayed*.

a *penalized beta*.

## Discussion

The main aims of this study were to assess the prevalence of burnout among health professionals during the COVID-19 pandemic and to evaluate its predictors. Results show that severe levels of burnout and psychopathological symptoms had high prevalence, and that the work-related and psychological factors associated with the necessity to cope with the COVID-19 emergency increase the risks of negative psychological consequences.

Moderate to severe levels of emotional exhaustion and reduced personal accomplishment were present in more than 60% of the sample, and moderate to severe levels of depersonalization in more than 25% of the sample. These results are novel since, to our knowledge, prevalence of burnout among health professionals during the COVID-19 pandemic has not been studied before. More importantly, these results have clear implications for both the professionals' health and efficiency of the health care systems. From the perspective of the professionals, burnout is associated with increased risks of both physical and psychological long-term detrimental consequences (Salvagioni et al., 2017). From the perspective of the health care systems, burnout is associated with increase in sick leave, absenteeism, job withdrawal and poor work efficiency (Salvagioni et al., 2017). Given the potential extended duration of the pandemic (Giordano et al., 2020), the negative impact of the high prevalence of burnout might worsen and reduce the capacity of health systems to cope with the increased demand of care that is likely to occur both in the short- and in the long-term (Boukhris et al., 2020; Leocani et al., 2020; Liebensteiner et al., 2020).

Analysis of burnout was complemented with the assessment of other psychological disorders. This helped to overcome the limitations of the cutoffs of burnout measures (Schaufeli and Van Dierendonck, 1995; Bianchi, 2015). In particular, prevalence of clinical levels of depression, anxiety, stress was higher than 25% in our sample. A previous rapid review with meta-analysis on 12 studies performed in China and one study performed in Singapore found that anxiety, depression and insomnia prevalence among health professionals during the COVID-19 outbreak was 23.2, 22.8, and 38.9%, respectively (Pappa et al., 2020). Taken together, these findings confirm that the impact of the pandemic on the health professionals' psychological health is massive. The respondents also showed very high levels of state anxiety, which might suggest the presence of a pervasive state of tension that could help the development or worsening of burnout and psychological distress symptoms. In addition, more than one participant out of four also showed post-traumatic symptoms. Previous studies performed after the SARS pandemic show conflicting results regarding the presence of post-traumatic symptoms among health workers, potentially attributable to the preparedness to face the emergency (Chan and Huak, 2004; Lee et al., 2007). The high prevalence of these symptoms that was found in this study might suggest the lack of preparation to face the emergency, and that the COVID-19 emergency has the potential to trigger traumatizing experiences for health professionals.

The regression models clearly show that the increased workload, the constant contact with COVID-19 patients and the psychological aspects related to their care are related to the levels of burnout. On the one hand, this calls for political and organizational decisions. Although the main focus of health care systems is on minimizing transmission, treating the infection, and saving lives, attention should be made to reduce the work-related burden on health professionals. Attention should be focused on promoting positive and protective strategies to cope with the emergency developed with the support of a dedicated psychologist.

On the other hand, these results show that presence of previous psychological comorbidities, fear of infection and feelings of isolation due to perceived lack of support from friends should be taken into account by interventions aimed at preventing the development of burnout in health professionals.

Timely recognition of this problem should help implement adequate prevention or rehabilitation strategies. In their review, Wiederhold et al. (2018) highlight that a successful intervention for burnout should take into account the broad range of causes and should incorporate a variety of different therapeutic tools. For this reason, it is necessary to promote monitoring of the health status, including mental health, of health workers during these moments of crisis. Several strategies could be implemented during and after the emergency to support health professionals working with COVID-19 patients, which include work-hour regulation programs, the implementation of strategies to reduce the pressure of difficult decision-making, planning official and unofficial rewards, providing individual or group psychological support programs, promoting focus groups to advance proposals for improvement of the organization of the work, providing individual and group skill training programs as well as online cognitive behavior therapy or mindfulness-based therapy (Ho et al., 2020).

The main limitation of this study is the heterogeneity of the sample. Although the inclusion of health professionals with different occupations and working in different wards allowed to provide a more complete picture of the impact of the pandemic, the variety of the respondents' characteristics. In addition, similarly to other studies performed during epidemics (Maunder et al., 2006; Lee et al., 2007), the respondent rate was low, indicating the risk of the auto-selection of the sample. Moreover, the cross-sectional nature of this study limits our understanding of the risk factors of burnout and suggests that longitudinal studies are needed for this purpose. Finally, the assessment of burnout, psychological distress and post-traumatic symptoms was performed using self-reported instruments which were not confirmed by medical records or specialistic evaluations.

In conclusion, this study shows that health professionals have a high risk of incurring in burnout or psychological conditions due to the COVID-19 pandemic. Continuous monitoring and timely treatment of these conditions is needed to preserve the professionals' health and to enhance the healthcare systems preparedness to face the medium- and long-term consequences of the outbreak.

## Data Availability Statement

The datasets presented in this article are not readily available because due to the nature of this research, participants of this study did not agree for their data to be shared publicly, so supporting data is not available. Requests to access the datasets should be directed to e.giusti@auxologico.it.

## Ethics Statement

The studies involving human participants were reviewed and approved by Comitato Etico dell'Istituto Auxologico Italiano. The participants provided their electronic informed consent to participate in this study.

## Author Contributions

EG, EP, CS, and CM were responsible for drafting the manuscript. All authors critically revised it for important intellectual content, gave final approval to the finished manuscript, and agreed to be accountable for all aspects of the work.

## Conflict of Interest

The authors declare that the research was conducted in the absence of any commercial or financial relationships that could be construed as a potential conflict of interest.
